# An Operational Hybrid SIEM Framework for OT Anomaly Detection †

**DOI:** 10.3390/s26103155

**Published:** 2026-05-16

**Authors:** Jaafer Rahmani, Salva Daneshgadeh Çakmakçı, Kai Oliver Detken, Axel Sikora

**Affiliations:** 1 Institute of Reliable Embedded Systems and Communication Electronics (ivESK), Offenburg University of Applied Sciences, 77652 Offenburg, Germany; axel.sikora@hs-offenburg.de; 2 Faculty of Engineering, University of Freiburg, 79110 Freiburg, Germany; 3 DECOIT GmbH & Co. KG, 28215 Bremen, Germany; daneshgadeh@decoit.de (S.D.Ç.); detken@decoit.de (K.O.D.)

**Keywords:** SIEM, anomaly detection, multi-layer telemetry, autoencoder, edge computing, operational technology, industrial Internet of Things, MITRE ATT&CK

## Abstract

Security monitoring in Industrial Internet of Things environments requires telemetry that spans Information Technology (IT) and Operational Technology (OT) network layers, and most public datasets capture only one such view. We describe a design pattern for hybrid Security Information and Event Management (SIEM) deployments in OT environments (rule-based detection plus edge-deployed machine learning anomaly detection writing into a shared index) and validate it on a Modbus/Jetson/Elastic instance. The pattern is platform-independent: any rule engine that exposes a query language and any edge device with adequate memory headroom can host an instance, and the paper documents the architectural choices that make this portability concrete. The validated instance comprises 27 rules in Kibana Query Language mapped to MITRE Adversarial Tactics, Techniques, and Common Knowledge, plus a CNN-BiLSTM autoencoder on a Jetson Orin Nano that reaches a true positive rate of 1.000 at the 98th-percentile validation threshold and 0.997 at the 99.5th-percentile threshold on a 9997-flow held-out attack partition. Runtime behaviour on the edge hardware is characterised under steady state and adversarial burst, including the queue-wait regime that dominates tail latency. A self-contained calibration step projects rule and model evidence onto a common scale for downstream fusion.

## 1. Introduction

Security monitoring in Industrial Internet of Things environments requires detection capabilities that span both Information Technology (IT) and Operational Technology (OT) network layers [1]. Traditional Intrusion Detection Systems and Security Information and Event Management solutions face three challenges in this context. First, the heterogeneity of data sources (network flows, host events, protocol transactions) demands multi-modal data fusion rather than analysis of isolated telemetry streams. Second, resource-constrained edge devices in OT networks limit the complexity of detection models that can be deployed close to the data source. Third, the convergence of IT and OT networks exposes industrial systems to multi-stage attacks that require correlation across network boundaries [2,3].

Existing IIoT anomaly detection datasets (Table 1) provide valuable benchmarks but typically capture only isolated views of network activity. IoTID20 [4] and CICIoT2023 [5] focus on flow-level features without protocol-specific data. The TON_IoT dataset [6] includes telemetry and OS logs but lacks Modbus or MQTT protocol data. CIC-APT-IIoT [7] addresses Advanced Persistent Threat scenarios but provides provenance logs rather than multi-layer SIEM telemetry. None of these datasets unify network, host, and protocol data within a SIEM context, limiting their utility for evaluating hybrid detection architectures.

This paper describes the framework as a design pattern, validated by one operational deployment on a physical OT testbed. The pattern has three load-bearing decisions, each separately portable to other stacks: (i) a rule path written in the query language the SIEM platform exposes for detection content, so rule provenance can be audited without crossing system boundaries; (ii) an ML path that runs on hardware co-located with the protocol whose normal distribution it learns, so detection latency stays inside the protocol’s polling cycle; (iii) a single backing index that both paths read and write, so joint analysis is one query rather than a cross-system join. A practitioner whose stack is Wazuh + Suricata + a different edge device can apply the same three decisions; the Modbus/Jetson/Elastic instance reported here is the form of evidence we offer for that pattern, not a constraint on it.

Section 2 situates the work against the dataset, hybrid-architecture, and edge-deployment threads of prior work and ends with the contributions claimed here. Section 3 describes the framework architecture. Section 4 presents the SIEM detection rules. Section 5 covers the edge deployment of the Modbus autoencoder. Section 6 reports the operational validation. Section 7 discusses findings and limitations, and Section 8 concludes.

## 2. Related Work

Prior work on hybrid intrusion detection for industrial environments spans three threads that motivate the present paper. The dataset thread (Section 2.1) supplies the raw telemetry against which detection is benchmarked. The hybrid-architecture thread (Section 2.2) combines rule-based and machine learning detection but rarely reports a single operational deployment in which both styles operate against the same multi-modal telemetry. The edge-deployment thread (Section 2.3) establishes the hardware constraints that any deployed detector must respect. Each subsection surveys one thread, identifies the gap the thread leaves open, and states how the present paper resolves that gap.

### 2.1. IIoT Security Datasets

Table 1 compares publicly available IIoT/IoT security datasets. The comparison focuses on data modalities, attack coverage, and limitations relevant to hybrid detection.

Gap: No public dataset provides network, host, and Modbus-protocol telemetry inside a shared SIEM index for IIoT environments. This limits evaluation of hybrid detection systems that need to correlate across layers with the same correlation semantics an operational SIEM provides. Our framework generates such datasets by integrating NetFlow, Auditd, and Zeek data through Elastic Agents, with SIEM rule alerts providing an additional correlated data layer (Section 3).

### 2.2. Hybrid Detection Architectures

Hybrid detection systems combine signature-based and anomaly-based approaches to address their individual limitations. SIEM platforms apply rule-based detection against known attack patterns, while machine learning models identify behavioural deviations without prior knowledge of attack signatures. The two detection styles answer complementary questions: rules answer does this match a known-bad pattern, while models answer does this deviate from the learned normal. Neither question subsumes the other, and a deployment that carries only one of them leaves the other unanswered. Recent work has demonstrated the effectiveness of the combination in several contexts.

Sheeraz et al. [11] developed a SIEM correlation engine using the Hyperscan pattern matching library, achieving parallel log scanning across multiple detection rules with reduced latency. Their work demonstrates the scalability of rule-based SIEM detection at high event rates but does not integrate ML-based anomaly detection, so the complementary question property above is not exercised in their deployment.

Ensemble approaches combining deep learning with traditional machine learning have achieved detection accuracies exceeding 99% on benchmark datasets [12]. These results confirm that diverse classifier families can be usefully combined when their error profiles are decorrelated. The benchmarking is conducted on static pre-labelled datasets, so the operational question of how the ensemble ingests live telemetry and how its decisions surface to a SIEM operator is answered by deployments such as the one described in this paper, not by the ensemble study itself.

Federated learning frameworks have shown promise for distributed IIoT environments. Reis [13] proposed Edge-FLGuard for 5G-enabled IoT ecosystems, demonstrating sub-20 ms inference latency on edge devices. Federated learning addresses privacy and scalability properties that are orthogonal to the SIEM-integration question addressed in the present paper; the two lines of work compose cleanly (federated model updates could feed the edge-deployed detector described here without changing its interface to the SIEM index).

SIEM distributions that bundle rule-based detection with machine learning augmentation are the natural points of reference for the present framework. Several mature distributions support user-authored detection content out of a public ruleset repository: Wazuh [14] (open-source, GPL-licensed rule files), the Elastic Stack used here [15] (KQL detection rules at https://github.com/elastic/detection-rules (accessed on 19 April 2026)), and Splunk Enterprise Security [16] (SPL correlation searches with detection content distributed via the Enterprise Security Content Update repository). Exabeam Fusion [17] ships behavioural-analytics models whose detection logic is vendor-proprietary and falls outside this group. What distinguishes the framework reported here from the user-authorable-rule SIEMs is a combination of axes that none of them ships out of the box: native Modbus protocol awareness in the rule path, an edge-deployed protocol-specific autoencoder co-located with the OT network, and a single backing index that carries both rule matches and model anomaly events as first-class documents. Table 2 summarises the resulting deployment-axis differentiation.

Gap: Existing hybrid architectures either combine different ML models without SIEM integration or deploy SIEM rules without ML augmentation; few published systems report a single deployment in which both detection styles operate against the same multi-modal telemetry. The present paper describes the testbed deployment in which a MITRE-mapped rule set and an edge-deployed autoencoder operate against a common Elasticsearch index (Section 4 and Section 5).

### 2.3. Edge Deployment for OT Security

Deploying ML models on resource-constrained edge devices in OT environments presents specific challenges: limited memory (shared CPU/GPU on ARM SoCs), real-time inference requirements, and operational safety constraints that prohibit concurrent training and inference [18]. The Jetson Orin Nano, with 8 GB shared RAM and an ARM Cortex-A78AE processor, represents a realistic edge deployment target for ICS environments: it sits below the power envelope of a rackmount server yet above the inference budget of a microcontroller, which is the gap where Modbus polling windows (1–10 s) coincide with useful inference latencies.

Recent work on intrusion detection for resource-constrained IoT settings has demonstrated that lightweight neural anomaly detectors with small parameter budgets can achieve competitive detection performance while fitting within memory constraints [19]. The operational implication is that a parameter-budget-aware detector design (architecture chosen so the model fits alongside other edge-resident processes) is a first-class deployment concern, not a post hoc optimisation. Our Modbus autoencoder (∼50,000 parameters) is sized for that deployment class and for the co-residency pattern described in Section 5, where the same SoC also hosts the feature extraction pipeline and (on the testbed) a quantised LLM for report generation.

Gap: We are not aware of ICS anomaly detection publications that report inference latency and operational constraints (e.g., no concurrent training and inference) measured on the actual edge hardware on which the detector is deployed. We provide this validation in Section 5 and Section 6.

### 2.4. Contributions of This Extension

The three gaps identified above motivate the contributions of this journal extension:

1. **Lead: a running instance of the two-path hybrid SIEM architecture on physical OT hardware, with an empirical finding on edge-SoC contention.** A running instance of parallel rule-based and machine learning detection over a shared SIEM index is reported on physical OT hardware, with its runtime behaviour quantified on a shared-memory Jetson Orin Nano. Under mixed benign and adversarial request workloads, tail latency on the critical detection path is set by queue-wait time at the pipeline’s concurrent inference cap (two simultaneous detections, sized for out-of-memory safety on the 8 GB shared-memory SoC): the 99th-percentile latency rises from 0.72 s at the steady-state operating point to 10.07 s under adversarial burst, with throughput dropping from 7.33 to 0.58 requests per second (Section 6.2). This is the design constraint that the two-path architecture must satisfy under live conditions, and it closes the edge deployment gap of Section 2.3.
2. **Supporting: a 27-rule MITRE-mapped detection set, justified by technique.** OT-layer rules are authored from scratch because Elastic Security ships no Modbus/TCP detection content out of the box; each OT rule is justified by the ICS technique it targets. IT-layer rules are selected from the public Elastic Detection Rules repository (approximately 800 prebuilt rules across the MITRE Enterprise matrix), restricted to the subset whose techniques appear in the IT-to-OT kill chains the testbed exercises (Section 4, full set in Appendix A). The headline edge-deployed autoencoder detection result for this use case is reported in Section 5.1; the contribution scoped here is the rule set itself and its integration with the edge-deployed model over the shared index, which closes the hybrid architecture gap of Section 2.2.

In the following sections, we present a high-level operational overview of the testbed and the edge-deployed autoencoder rather than their design details: the testbed hardware, the dataset construction protocol, and the per-technique sensitivity of the model are documented in the companion submission and are not re-derived here. Per-technique evaluation of the autoencoder is therefore not the focus of this study; the framework-level concerns (rule provenance, calibration, edge runtime behaviour, two-path coverage) are.

**Scope and external dependencies.** Three artefacts that this paper consumes as inputs are documented in separate publications and not re-derived here: the physical OT testbed (PLC and SCADA hardware, Modbus benign and attack capture, MITRE-mapped session-correlation layer), the per-technique sensitivity of the autoencoder model, and the CALDERA kill-chain definitions used to exercise the deployment, all in [20]; the PROFINET detector that also rides the pipeline, in [21]; and the MITRE CALDERA adversary emulation framework itself [22]. Subsequent sections refer to “the testbed” and “the autoencoder” by name; the bibliographic anchors are kept consolidated to this paragraph because the companion publications are still under review.

## 3. Framework Architecture

**Figure 1:**
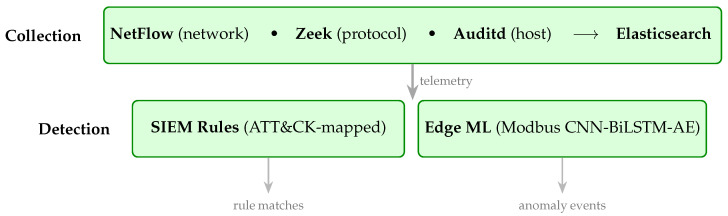
SIEM-based multi-layer detection framework. The Collection layer (Elastic Agents forwarding NetFlow, Zeek, and Auditd events into Elasticsearch) aggregates multi-modal telemetry into a central index. The Detection layer runs the SIEM rules and the edge-deployed Modbus autoencoder over that index and produces two detection streams available to a downstream consumer.

The framework is a multi-layer security monitoring system for Operational Technology networks. Its purpose is to consolidate telemetry from heterogeneous sources into a single index and run two complementary detection paths (rule-based and machine learning) over that index. The framework itself, rendered in Figure 1, has two layers, and it operates on the physical OT testbed (the SCADA/PLC/Modbus environment and the autoencoder model artefact). A Collection layer consolidates multi-modal telemetry into a centralised SIEM index. A Detection layer runs two parallel paths against that index: a rule-based path (the Elastic detection rules of Section 4) and a machine learning path (the edge-deployed Modbus autoencoder of Section 5). A downstream consumer that combines the two detection streams into a single decision is rendered in the figure as a forward handoff; the present paper specifies the events that arrive at that handoff and not the combination logic.

### 3.1. Architectural Choices

Three architectural decisions shape the framework as described in Figure 1 and merit explicit justification. First, the two detection paths run in parallel rather than in series. A serial design (rule match as a pre-filter into ML, or ML score as a pre-filter into rule match) forces one path to act as a gate for the other, which would silence either the rule-based evidence on ML-silent flows or the ML evidence on rule-silent flows. Parallel operation lets each path emit evidence on every flow it finds relevant, so events one path misses remain visible to the other. Second, both paths read from a single shared Elasticsearch index rather than from siloed per-path stores. The shared index gives both paths the same time-synchronised view of the telemetry and lets a downstream consumer temporally correlate rule matches with model detections without bridging two different data stores or reconciling two different clocks. Third, rule matching runs in situ on the live Elastic Security alert index that operators already query through Kibana, rather than on a parallel queue. This keeps rule behaviour inspectable in the same interface operators use for day-to-day analysis and avoids a parallel rule engine whose behaviour would need to be independently validated against Elastic’s.

### 3.2. Relationship to the Testbed

The framework consumes one input stream from the testbed: the physical SCADA/PLC/Modbus environment, instrumented with Elastic Agents that forward telemetry into the framework’s Elasticsearch index. The framework produces two output streams for a downstream consumer: (1) SIEM rule matches produced by the Elastic detection rules (Section 4) against the centralised index; (2) per-flow anomaly events produced by the Modbus autoencoder (Section 5), each carrying an anomaly score and, where available, MITRE ATT&CK for ICS technique enrichment from the testbed’s session correlation layer.

## 4. SIEM Detection Rules

This section describes the rule-based detection path: the structure of the rule set that runs on the testbed, its coverage of MITRE tactics, and the way operator-driven false positive management is handled when a rule fires on legitimate traffic.

The rule set is justified by technique. Each rule instantiates a specific MITRE ATT&CK technique entry (Enterprise or ICS matrix), with the technique description and its observed procedures acting as the specification for the query pattern; the tactic coverage reported in Table 3 is a consequence of this technique-first authoring. The two layers of the rule set were assembled by different routes. OT-layer rules are authored from scratch in this work because Elastic Security ships no Modbus/TCP detection content out of the box; each OT rule is therefore justified by the ICS technique it targets, and the rule queries are calibrated against the CALDERA adversary chains, whose ability logs provide reference traces that a candidate rule must match without matching the benign operation captured on the testbed between campaigns.IT-layer rules are selected from the public Elastic Detection Rules repository (https://github.com/elastic/detection-rules (accessed on 19 April 2026)), which ships approximately 800 prebuilt rules across the MITRE ATT&CK Enterprise matrix; the subset enabled here is restricted to techniques that appear in the IT-to-OT kill chains the testbed exercises (T1110.003, T1098, T1003, T1021.002, T1087.002, T1482, T1484.001 are representative).

The rule set is authored in Kibana Query Language (KQL) [15], the query language of the Elastic Security platform that hosts the deployment. KQL is one of three languages the Elastic ecosystem exposes (alongside Event Query Language and Query DSL); KQL is selected for the rules described here because it is the language Elastic Security exposes for detection-rule authoring, which keeps rule semantics aligned with the platform that executes them. Its rules break down into two semantic categories, tagged by the network layer they target (IT versus OT). OT-tagged rules target Modbus/TCP-specific patterns (denial-of-service and broadcast abuse, safety-system tampering, forced output manipulation, unauthorised writes from non-SCADA hosts, covert channel signalling, mass-read exfiltration, request flooding, and unit-ID/function-code/register-space scanning). IT-tagged rules target enterprise ATT&CK techniques reachable through host and network telemetry on the IT side of the testbed (password spraying, UAC bypass, GPO and network-share discovery, credential-file access, command-history clearing, remote-system discovery, registry-hive and autologon-credential access, domain-account enumeration, and SMB lateral movement). Each rule carries a query pattern, a severity level, a MITRE ATT&CK tactic-and-technique mapping, and an exclusion list for known benign sources. The 27 rules distribute across severity bands (5 critical, 15 high, 5 medium, 2 low) so that alert-queue triage has an explicit priority signal rather than uniform urgency across the set. Table 3 summarises the rule set’s tactic coverage across the MITRE ATT&CK Enterprise and ICS matrices.

False positives are managed through per-rule exclusion lists, which are an operator-maintained allow-list of network-flow attributes (source IP, destination IP, function code, time window) that the rule engine excludes from a rule’s match set before issuing an alert. Per-environment exclusion lists are the established industry norm for rule-engine SOCs: a published rule ships in its default state, and once it produces enough false positives on the local baseline (the SCADA poller’s normal traffic, scheduled scanners, configuration-management hosts) the operator authors an exclusion clause to suppress the legitimate triggers without weakening the rule against an attacker. The mechanism applies to any rule engine that exposes a query language: in the deployment reported here it is realised as a NOT clause appended to the KQL body of the affected rule, but the same approach applies to Wazuh decoders, Suricata signatures, or Splunk search-time eval expressions. An illustrative case is the “unauthorised write from non-SCADA host” rule, which initially triggered on setpoint writes from the legitimate SCADA poller. Two exclusions resolve this without weakening the rule against an attacker: a source-IP exclusion for the poller’s address suppresses the benign trigger, and a complementary destination-IP scope clause keeps the rule active on writes addressed to PLCs the poller is not authorised to reach (so a misrouted poller request still alerts). The exclusion is not unconditional whitelisting. The rule engine and the autoencoder are separate detection systems, but they write into the same Elasticsearch index: a rule match on the spoofed write would never fire because the source IP is allow-listed, while the same network event is independently scored by the autoencoder on payload-shape features (function code, register count, request-response latency, request rate; Section 5.1) that are independent of source-IP attribution, and any anomaly the model flags is written to the same index as a separate document. From the analyst’s perspective the spoof attempt surfaces on the ML path even though it bypasses the rule path, because both detectors publish into a single backing store. The two-path architecture therefore turns each rule’s exclusion list into an artefact the ML path can cross-check, rather than a blind spot. Rules that trigger on legitimate behaviour during the testbed’s benign windows are addressed with exclusions of the same shape as the illustrative case above.

## 5. Edge Deployment of the Modbus Autoencoder

This section describes how the machine learning half of the hybrid detection path is deployed. The model is an autoencoder: a neural anomaly detector trained on benign Modbus traffic to reconstruct its input, so that anomalous flows produce elevated reconstruction error. Its architecture is a one-dimensional convolutional network combined with a bidirectional Long Short-Term Memory network, sized at approximately 50,000 parameters so that it fits within the Jetson Orin Nano’s 8 GB shared CPU/GPU memory alongside the feature-extraction pipeline. The present section covers how the model is integrated with the SIEM layer of the framework.

The autoencoder runs on a Jetson Orin Nano co-located with the SCADA network. For every flow it scores, it produces a record with a reconstruction error (mean squared error between the autoencoder’s input and its output), an anomaly score derived from that error, and, where available, MITRE ATT&CK for ICS technique enrichment attached by the upstream session-correlation layer. The record is written into the framework’s Elasticsearch index so that rule-based and machine learning detection events are available to a downstream consumer as entries in the same index.

### 5.1. Use-Case Dataset, Training, and Threshold Selection

For the use case validated here, the autoencoder is trained on the Modbus traffic captured on the testbed described above. This subsection summarises the dataset shape, the training procedure, and the threshold-selection rule as they apply to the integrated deployment so that the present paper is independently assessable; the full per-technique evaluation table and the dataset-construction protocol are reported in the same reference.

**Dataset.** The training and evaluation set comprises 40,000 benign Modbus flows (the SCADA poller’s normal read/write cycle on the Raspberry-Pi PLCs of the testbed) and 9997 attack flows (CALDERA-orchestrated MITRE ATT&CK for ICS techniques covering the Collection, Discovery, and Impair Process Control tactics). Each flow is encoded as an eight-feature vector: function code, register count, payload-length deviation, request-response latency, request rate, function-code read fraction, function-code transition entropy, and time-of-day. The benign partition is used for autoencoder fitting; the attack partition is held out for evaluation only.
**Training.** The CNN-BiLSTM autoencoder (∼50,000 parameters) is trained with mean-squared reconstruction error on the benign partition, optimised with Adam, with early stopping on a held-out 10% validation split. The training loop runs off the critical path on the Jetson Orin Nano: model fitting and online inference do not run concurrently, which avoids the OOM regime described in Section 6.2.
**Threshold selection.** The deployment uses two anomaly thresholds derived from the held-out validation reconstruction error distribution: $\tau_{p98}=1.263$ (98th percentile) and $\tau_{p99.5}=1.345$ (99.5th percentile). Reconstruction error above τ is reported as an anomaly with the corresponding confidence level. The two thresholds let an operator trade sensitivity against false positive volume without retraining the model: $\tau_{p98}$ is the high-recall setting used during incident triage, $\tau_{p99.5}$ is the low-FPR setting used during steady-state monitoring. The validation reconstruction error distribution has mean 0.79 and standard deviation 0.24; observed attack-class mean reconstruction errors are in the range 2.37 to 2.49 for the techniques exercised on the testbed, so both thresholds sit well inside the benign attack margin.
**Headline use-case detection result.** On the held-out 9997-flow attack partition the autoencoder reaches an overall true positive rate of 1.000 at $\tau_{p98}$ and 0.997 at $\tau_{p99.5}$ (Table 4). From the framework’s perspective, this result confirms that the ML detection events that arrive at the SIEM index carry real signal for the techniques the rule path also covers.
**Operational false positive characterisation.** For an anomaly detector the true positive rate alone does not bound operational usability: an operator also needs to know how often the detector raises an alert on benign traffic and how often a raised alert is correct. Both thresholds are derived as quantiles of the benign validation reconstruction error distribution (Section 5.1), so the false positive rate on the benign validation partition is fixed by construction: $\mathrm{FPR}(\tau_{p98})=2.0\%$ (80 of 4000 benign validation flows above τ) and $\mathrm{FPR}(\tau_{p99.5})=0.5\%$ (20 of 4000). The benign validation partition is held out from autoencoder fitting (it is used only for early stopping and threshold derivation), so the construction yields the operational FPR estimate on the benign distribution rather than a training-set FPR. Table 5 summarises the resulting operational metrics: false alarm rate per hour at the steady-state throughput observed on the Jetson Orin Nano (7.33 requests/s, Section 6.2), precision on the combined held-out evaluation partition (4000 benign + 9997 attack flows), and $F_{1}$ at each threshold. Precision is reported on the held-out evaluation partition’s class balance; operational precision in a deployment whose benign/attack ratio differs from this partition can be re-derived from the reported FPR and TPR. Both thresholds give a precision above 0.99 on the evaluation partition, with $\tau_{p99.5}$ trading 0.003 of recall for a four-fold reduction in false alarm rate; the choice between them is the standard operator trade-off between triage sensitivity and steady-state alert-queue volume.

The FPR figures above are calibration-set FPRs by construction. A practitioner moving the deployment to a site with a different benign Modbus baseline should re-derive both thresholds from that site’s benign validation distribution, since the construction (percentile of the validation reconstruction error distribution) is what fixes the operational FPR and not the absolute reconstruction error values reported here. The calibration step of Section 5.2 carries this site-dependence forward into the downstream score the SIEM index consumes.

### 5.2. Anomaly-Score Calibration

Raw reconstruction errors vary in magnitude across models and training runs, so the score field that arrives at the SIEM index is a calibrated value rather than the raw reconstruction error. The calibration is a one-pass min–max projection against the held-out validation reconstruction error distribution of the deployed model. Let *e* be the raw reconstruction error of an incoming flow and $E_{v}=\left\{e_{1},\ldots ,e_{n}\right\}$ the reconstruction errors of the held-out 10% validation split (Section 5.1, n=4000 on the use-case dataset). The calibrated score s∈[0,1] is

(1) $$s\;=\;\min\;\left(1,\;\max\;\left(0,\;\frac{e-q_{p}\left(E_{v}\right)}{q_{q}\left(E_{v}\right)-q_{p}\left(E_{v}\right)}\right)\right),$$

with $q_{p}\left(E_{v}\right)$ and $q_{q}\left(E_{v}\right)$ the *p*th and *q*th percentiles of the validation distribution. The deployment uses p=50 (median) as the lower anchor and q=99.5 as the upper anchor, so a calibrated score of 0.5 corresponds to the operational $\tau_{p98}$ alerting threshold and a calibrated score of 1.0 saturates at the validation distribution’s 99.5th-percentile tail. The two anchor percentiles are stored alongside the model artefact so that downstream consumers (and any second-pass fusion stage) read the calibration coefficients from the artefact rather than recomputing them per request. Because the calibration anchors are derived from the same benign validation split that fixes the alerting thresholds $\tau_{p98}$ and $\tau_{p99.5}$, the calibrated score and the threshold flag carry consistent semantics across model versions: a score of ≥0.5 corresponds to a $\tau_{p98}$ alert and a score of 1.0 corresponds to the $\tau_{p99.5}$ alert at any model version, regardless of the absolute scale of the underlying reconstruction error distribution. A downstream consumer that combines model detections with rule matches therefore reads model and rule severity on a common [0,1] scale.

## 6. Operational Validation

This section reports the operational validation of the framework as deployed on the testbed. It describes the experimental setup (Section 6.1), the runtime behaviour of the deployment on the edge hardware (Section 6.2), and the activity of the two detection paths when the testbed is exercised by a set of adversary-emulated kill chains (Section 6.3).

### 6.1. Experimental Setup

The deployment runs on the physical testbed. The collection pipeline and the edge autoencoder run on a Jetson Orin Nano (8 GB shared RAM, NVIDIA, Santa Clara, CA, USA); the SIEM infrastructure (Elasticsearch, Kibana, Fleet) runs on a dedicated server. All rules described in Section 4 and the Modbus autoencoder are active throughout.

A detection request in this paper is one HTTP invocation of the deployment’s detection endpoint carrying a single Modbus flow record encoded as the eight-feature vector of Section 5.1. The endpoint runs the protocol router, dispatches the flow to the Modbus autoencoder, and returns the per-flow score and label.

Workload is generated by a closed-loop driver that maintains a fixed target concurrency by issuing a new request as soon as a previous request completes. Two driver instances run in parallel during the interference condition: one issues five concurrent requests carrying benign Modbus flows (the critical path) and the other issues eight concurrent requests carrying attack-traffic Modbus flows (the interference path). The critical path corresponds to the steady-state operational workload (the SCADA poller’s flow under benign conditions); the interference path corresponds to the additional workload imposed when the deployment is also asked to score adversarial traffic during an active campaign. Throughput and latency are reported on the critical path only; the interference path is treated as background load that contests the same memory and inflight-slot resources.

### 6.2. Runtime Behaviour on the Edge Hardware

The edge deployment is exercised with two request workloads on the Jetson Orin Nano, each for 120 s with 20 s warmup. The first workload issues five concurrent benign detection requests to the framework and reports the resulting throughput and latency as the operating point of the deployment at a representative steady-state load. The second adds eight concurrent requests carrying attack-traffic payloads, which characterises the deployment’s behaviour when the edge device is also processing workload on the interference path. Table 6 summarises the observed values.

The SCADA polling interval sets the effective latency budget for edge anomaly scoring: a detection event must land before the next poll arrives for it to inform the operator within the same supervisory cycle. At steady state the 99th-percentile latency of 0.72 s is well inside the 1–10 s Modbus polling window typical of industrial environments. Under concurrent attack-payload interference the 99th-percentile latency rises to 10.07 s and throughput drops from 7.33 to 0.58 requests per second, leaving tail latency at the upper edge of the polling window. The detection pipeline enforces a concurrent-inference cap of two simultaneous detections to prevent out-of-memory faults on the 8 GB shared CPU/GPU SoC; with five critical-path and eight interference requests competing for the two inflight slots, queue-wait time dominates per-request service time, which is what drives the order-of-magnitude tail-latency rise. For the deployment evaluated here, a request-scheduling layer that reserves inflight capacity for the critical path is what keeps tail latency inside the polling window under adversarial burst conditions. This function lives in the detection-path scheduling logic, not in the collection layer described here.

The queue-wait explanation above addresses the resource-contention component of the observed degradation. In a hybrid deployment, interference can also arise from interactions between the rule path, the ML path, and the SIEM index that hosts both: rule-level interference between automation rules has been formalised for IoT automation systems by Cimino and Deufemia [23], and a hybrid IDS in an IIoT environment carries the broader integration concerns surveyed in [18]. On the deployment reported here, the two detection paths read from the same index and write back into it, but they do not query each other’s intermediate state, so cross-path interference at the rule-engine level is structurally avoided; what remains is the resource-contention component above. Cross-path interaction effects (e.g., correlated alert storms triggering rule re-evaluations) would surface only in deployments that wire the two paths into a feedback loop, which the architecture of Section 3 deliberately avoids.

### 6.3. Activity of the Two Detection Paths Across Kill-Chain Campaigns

When the testbed is exercised by the CALDERA-driven IT-to-OT kill chains, both detection paths of the framework produce events on the same Elasticsearch index. Per-chain detection counts, CALDERA-ability success rates, and tactic-coverage percentages are out of scope for this framework-level paper. From the framework’s perspective, the observation that matters is which detection path fires on which chain profile: Modbus-heavy chains drive the machine learning path, IT-focused domain-escalation chains drive the rule-based path, and low-volume stealthy chains produce activity below both paths’ thresholds. This is the division of labour the two-path architecture predicts, and it is visible under live deployment from the per-path event streams alone.

## 7. Discussion

### 7.1. Edge Deployment Lessons

The edge-hardware measurements of Section 6.2 point toward a single operating constraint observed in this use case on a shared-memory SoC: under mixed workloads the critical path’s tail latency is set by queue-wait time at the concurrent-inference cap. The evidence is the pair of measurements in Table 6: at five concurrent critical-path requests the 99th-percentile latency is 0.72 s, and adding eight interference requests pushes it to 10.07 s while per-request inference cost is unchanged. A concurrency cap sized for out-of-memory safety does not on its own bound tail latency once the request mix exceeds it. Three practices follow. First, memory budgeting on the shared CPU/GPU SoC must account for the combined footprint of the autoencoder, its feature preprocessing buffers, and any co-resident process; the framework treats model training as an off-critical-path activity for the same reason. Second, per-source temporal features carry state proportional to the number of active source IPs, which a bounded state table caps without disturbing the recency window used by the testbed’s session-correlation layer. Third, a request-scheduling layer that reserves inflight slots for the critical path is what keeps the edge deployment inside the polling window under adversarial burst conditions.

### 7.2. Operational Properties of the Two-Path Architecture

Running rule-based and machine learning detection as two paths over a common SIEM index gives the framework four deployment properties that are directly useful to an operator maintaining the system.

First, rule updates and model updates are independent maintenance activities. A new threat-intelligence report that motivates a new rule can be authored, tested, and rolled into the rule set without touching the edge-deployed autoencoder or its training pipeline; rule coverage is preserved across any later retraining of the model, since the two paths are evaluated on the same events but from different definitions of anomalous. Second, both paths write into the same Elasticsearch index with timestamps from the same time-synchronised Elastic Agent layer, so joint analysis across rule matches and model anomalies is an Elasticsearch query rather than a cross-system join with clock reconciliation. Third, new protocol coverage extends the ML path through addition rather than replacement: a new protocol-specific detector (for example, a PROFINET-specific detector for cyclic real-time traffic) can be added behind the protocol router without re-authoring the existing rule set or changing the SIEM infrastructure. Fourth, operator tooling stays inside the Elastic Security interface operators already use for day-to-day analysis, since rule matches and model-produced anomaly events are both first-class documents in the same index and surface through Kibana with the same filtering and correlation primitives.

### 7.3. Limitations

- **Single testbed, single model, single edge device.** The operational characterisation is conducted on one physical testbed, one autoencoder configuration, and one edge device (Jetson Orin Nano, 8 GB shared CPU/GPU). The mechanism the measurements expose (queue-wait time at the concurrent-inference cap dominating tail latency under burst conditions) is a property of shared-memory SoC scheduling rather than of the specific model, but the absolute throughput and latency numbers are device-specific and should not be extrapolated to other accelerator classes without measurement. Cross-site validation on additional ICS deployments and other edge accelerator classes would extend the evidence base for the deployment pattern.
- **Static rule-exclusion lists, not adaptive baselines.** False positive control on the rule path (Section 4) relies on operator-maintained per-rule exclusion lists keyed on source IP, destination IP, function code, and time window. The lists are static between operator updates and do not adapt to baseline drift (a new SCADA poller, a transient maintenance flow, a seasonal change in plant operation). The two-path architecture compensates for this in part, since the ML path carries the drift-sensitive component and is retrained on the new baseline rather than re-curated; future work on the rule path is to add a sliding-window baseline that auto-suggests exclusion candidates from rules that fire above a configured rate during operator-confirmed benign windows, with operator approval still required before the suggestion enters the rule file.
- **Model-update detection windows on the ML path.** The Jetson Orin Nano cannot run training and inference concurrently within its 8 GB shared CPU/GPU memory budget; consequently, the ML path is offline during retraining or fine-tuning of the autoencoder. The deployment mitigates this in three ways: (i) the rule path remains online throughout retraining and continues to alert on rule-matched activity, so the framework is not detection-blind during the window; (ii) retraining runs on a fixed cadence outside the SCADA polling cycles that produce the operational telemetry, with the new model artefact validated in shadow against the live stream before promotion (model promotion requires explicit operator action, not auto-promotion, so the new model never silently replaces the running one); (iii) a rule-only fallback mode for the affected protocol stays available throughout the window, so a Modbus campaign that lands during retraining still surfaces on the rule path. A dual-instance hot-swap, in which a second edge device hosts the candidate model while the production device serves traffic, would close the ML-path window entirely; this requires either a second Jetson per protocol or a quantised co-resident model, both of which we leave to future work.
- **Evaluation scope.** The CALDERA-driven kill-chain input in Section 6.3 exercises both detection paths as a deployment validation; the per-technique quantification of the autoencoder is out of scope for this framework-level paper.
- **Federated learning.** The current architecture supports local model updates but does not implement federated aggregation. Federated multi-site model updates remain future work.

## 8. Conclusions

We described a design pattern for hybrid SIEMs in OT environments and validated it on one operational instance. The pattern has three load-bearing decisions: a rule path in the query language the SIEM exposes for detection content (KQL on Elastic in this instance, with Wazuh decoders, Suricata signatures, and Splunk eval expressions as direct equivalents), an ML path co-located with the protocol whose normal distribution it learns (a CNN-BiLSTM autoencoder on a Jetson Orin Nano in this instance, with any edge device meeting the model’s memory budget as an equivalent host), and a shared backing index that carries rule matches and model anomalies as first-class documents (an Elasticsearch index in this instance, with any document store exposing the same correlation primitives as an equivalent backbone). Practitioners running a different rule engine or a different edge device can apply the same three decisions; the Modbus/Jetson/Elastic combination reported here is the form of the evidence, not the scope of the pattern.

On the validated instance, the autoencoder reaches a true positive rate of 1.000 at the 98th-percentile validation threshold and 0.997 at the 99.5th-percentile threshold on the held-out 9997-flow attack partition, and the runtime characterisation on the edge hardware shows the queue-wait regime that dominates tail latency under adversarial burst. The calibration step (Section 5.2) projects rule and model evidence onto a common scale so that downstream fusion does not need to reconcile heterogeneous magnitudes per artefact. Cross-site evaluation on additional ICS deployments and federated learning for multi-site model updates remain the immediate next steps.

## Figures and Tables

**Table 1:** Comparison of public IIoT/IoT security datasets. Our framework generates multi-layer datasets integrating network, host, and protocol data within a centralised SIEM.

Dataset	Data Features	Attack Types	Limitations
IoTID20 [4]	Flow data	D/DoS, MITM, scan	No host, protocol, or Modbus data
Kitsune [8]	Flow data	DDoS, MITM, injection	No host or protocol data
TON_IoT [6]	Telemetry, flows, OS logs	DoS, ransomware, web	No Modbus/MQTT protocol data
CICIoT2023 [5]	Flow data	D/DoS, recon, spoofing	No host or protocol data
CIC-APT-IIoT [7]	Flow and host logs	APT (multi-stage)	No Modbus protocol data; provenance only
X-IIoTID [9]	Flow, host, alerts	MITRE ATT&CK for ICS	No Modbus/MQTT protocol data
Edge-IIoTset [10]	Sensor, alerts, flows	DoS, MITM, malware	Limited host data
Our Work	NetFlow, Auditd, Zeek protocol events, SIEM alerts	MITRE ATT&CK-mapped	Single testbed; ongoing expansion

**Table 2:** Deployment-axis comparison between the framework reported here and two SIEM distributions that share its user-authorable-rule premise. *Native Modbus*: ships with Modbus/TCP-aware detection content out of the box. *Edge ML*: ML component runs on a device co-located with the OT network, not on the central SIEM tier. *Shared index*: rule matches and model-produced anomaly events live in the same backing index for joint querying.

Distribution	Rule Path	ML Path	Native Modbus	Edge ML	Shared Index
Wazuh [14]	host rules + Elastic	limited (anomaly module)	no	no	yes
Splunk ES [16]	SPL search-time	ML Toolkit (centralised)	no	no	yes
This framework	KQL on Elastic	CNN-BiLSTM-AE on Jetson Orin Nano	yes	yes	yes

**Table 3:** MITRE ATT&CK tactic coverage across the SIEM detection rule set. One rule may be tagged with multiple tactics, so the per-tactic counts can sum to more than 27.

Tactic	Rules
Discovery	6
Impact	5
Credential Access	4
Execution	2
Collection	2
Lateral Movement	2
Defense Evasion	2
Command and Control	1
Reconnaissance	1
Privilege Escalation	1
Persistence	1

**Table 4:** Use-case detection summary for the edge-deployed Modbus autoencoder, evaluated on the held-out CALDERA-orchestrated attack partition. TPR is reported at the two operational thresholds derived from the benign validation reconstruction error distribution. Tactic-level counts aggregate the per-technique breakdown: Collection covers T0802, Discovery covers T0846, Impair Process Control covers T0831 and T0836. Bold values in the last row indicate the aggregate result across all tactics on the full held-out attack partition.

MITRE ATT&CK for ICS Tactic	Samples	TPR @ $\tau_{p98}$	TPR @ $\tau_{p99.5}$
Collection	1548	1.000	1.000
Discovery	7025	1.000	1.000
Impair Process Control	1415	1.000	0.975
**Overall (held-out attack partition)**	**9997**	**1.000**	**0.997**

**Table 5:** Operational metrics for the edge-deployed Modbus autoencoder at the two thresholds, evaluated on the held-out partition (4000 benign + 9997 attack flows). FPR is fixed by construction at the chosen percentile of the benign validation reconstruction error distribution. The false alarm rate is the FPR projected onto the steady-state throughput observed on the Jetson Orin Nano (7.33 requests/s; Section 6.2). Precision =TP/(TP+FP); $F_{1}=2\;\mathrm{TP}/\left(2\;\mathrm{TP}+\mathrm{FP}+\mathrm{FN}\right)$.

Metric	$\tau_{p98}=1.263$	$\tau_{p99.5}=1.345$
True positive rate (TPR)	1.000	0.997
False positive rate (FPR)	0.020	0.005
False alarms per hour (steady state)	∼528	∼132
Precision	0.992	0.998
$F_{1}$	0.996	0.997

**Table 6:** Runtime behaviour of the deployment on the Jetson Orin Nano (8 GB shared RAM, 6 × Cortex-A78AE, NVIDIA Ampere GPU with 1024 CUDA cores). Each condition runs 120 s with 20 s warmup. *Steady state*: five concurrent benign detection requests. *With interference*: the same five benign requests plus eight concurrent attack-interference requests. Throughput reports successful requests per second on the detection path.

Metric	Steady State	With Interference
Throughput (requests/s)	7.33	0.58
Latency mean (ms)	684.1	9234.4
Latency p95 (ms)	709.9	10,065.2
Latency p99 (ms)	723.3	10,074.1
Latency max (ms)	1056.5	10,077.9
Modbus CNN-BiLSTM-AE	∼50 K parameters	

## Data Availability

Restrictions apply to the availability of these data. The data presented in this study were obtained within the BMWE-funded KISTE project (grant number KK5189606RG4) and are subject to a non-disclosure agreement between the project partners. They are available from the corresponding author on reasonable request and with the permission of the KISTE consortium.

## Appendix A. Full SIEM Rule Set

The 27 rules of the rule-based detection path are listed in Table A1, grouped by network layer (OT then IT), with the MITRE ATT&CK technique that each rule targets shown in a per-row column. Each rule carries a Kibana Query Language (KQL) query body, a MITRE ATT&CK technique mapping, and a severity level. The provenance of each layer (OT-layer rules authored from scratch against the Modbus specification and the ICS matrix; IT-layer rules selected from the public Elastic Detection Rules repository against the Enterprise matrix) is described in Section 4. Rule queries follow a common pattern of [event-type filter] AND [pattern check] AND NOT [exclusion list]; full query bodies are available in the project’s rule configuration. OT-tagged rules consume Modbus protocol events emitted by Zeek (e.g., zeek.modbus.function, zeek.modbus.request_subfunction_code); IT-tagged rules consume Windows process and event-log records forwarded by Elastic Agents (e.g., process.name, event.action); the port-502 sweep rule consumes network-flow telemetry from the destination-address field.

For each rule, Table A2 reports the trigger condition (the pattern in the telemetry that causes the rule to fire), the key telemetry fields (the indexed document fields the query reads), and the common exclusions (the operator-curated allow-list clauses that suppress legitimate triggers without weakening the rule against an adversary, per Section 4). The exclusions listed are the ones in force on the testbed deployment; per-environment exclusion lists are tuned to the local baseline (poller IPs, vendor maintenance windows, software deployment service accounts).

**Table A1:** Full SIEM rule set (27 rules, grouped by network layer with per-rule MITRE ATT&CK technique mapping). *Layer*: IT (enterprise-side telemetry: Windows process and event logs) or OT (Modbus protocol events from Zeek, plus OT-network flow telemetry). *Tech. (Technique Identifier)*: MITRE ATT&CK technique identifier (Enterprise for IT rules, ICS for OT rules). *Sev. (Severity)*: C = critical, H = high, M = medium, L = low. The aggregate tactic coverage of the rule set is summarised in Table 3.

#	Rule	Layer	Tech.	Sev.
1	Modbus DoS: broadcast or restart attack	OT	T1499	C
2	Modbus inhibit response: safety-system tampering	OT	T0816	C
3	Modbus execution: forced output manipulation	OT	T0855	C
4	Modbus command and control: covert channel	OT	T1071	H
5	Modbus collection: mass data exfiltration	OT	T0802	H
6	Modbus impair process control: critical-value manipulation	OT	T0831	H
7	Modbus DoS: request flood	OT	T1499	H
8	Modbus lateral movement: unit-ID scanning	OT	T1021	H
9	Modbus reconnaissance: function-code scanning	OT	T0846	H
10	Modbus unauthorised source: write from non-SCADA host	OT	T0831	C
11	Modbus unauthorised source: read from non-SCADA host	OT	T0802	H
12	Modbus kill chain: read-then-write sequence	OT	T0855	C
13	Modbus discovery: register-space mapping	OT	T0846	M
14	IT password spray: multiple failed logons	IT	T1110	H
15	IT UAC bypass via PowerShell module	IT	T1548	H
16	IT suspicious GPO update from command shell	IT	T1484	M
17	IT network-share discovery via command line	IT	T1135	M
18	IT scheduled task via PowerShell cmdlet	IT	T1053	H
19	IT file and directory discovery via command line	IT	T1083	L
20	IT credential-file access via command line	IT	T1552	H
21	IT command-history clearing	IT	T1070	M
22	IT remote-system discovery via ARP or net view	IT	T1018	L
23	IT credential acquisition via registry-hive dumping	IT	T1003	H
24	IT registry autologon credential extraction	IT	T1552	H
25	IT domain-account and group enumeration	IT	T1087	M
26	IT lateral movement via SMB admin share	IT	T1021	H
27	OT network-service scan: port-502 sweep	OT	T1046	H

**Table A2:** Per-rule trigger condition, key telemetry fields, and common exclusions for the 27-rule SIEM detection set. Row numbers match Table A1. Field names use the Elastic Common Schema (ECS) where applicable, with zeek.modbus.* for Modbus protocol events from Zeek and winlog.* for Windows event-log records.

#	Trigger Condition	Key Telemetry Fields	Common Exclusions
1	Broadcast unit ID (0) with function-code value indicating Restart Communications or Force Listen Only	zeek.modbus.unit_id, zeek.modbus.function	Vendor firmware-reload windows scoped to a source IP
2	Diagnostic function code (0x08) with sub-function Force Listen Only or Restart Communications Option	zeek.modbus.function, zeek.modbus.request_subfunction_code	Vendor maintenance source IPs during planned outages
3	Write Single Coil (0x05) or Write Single Register (0x06) targeting safety-tagged register ranges from a non-SCADA source	zeek.modbus.function, zeek.modbus.start_address, source.ip	SCADA poller source IP scoped to its authorised destination PLCs
4	Repeated reads of write-only registers, or elevated payload entropy on Modbus function codes that carry fixed-shape data	zeek.modbus.function, zeek.modbus.length, zeek.modbus.start_address	Telemetry-bridging hosts that legitimately re-encode register data
5	Read Holding/Input Registers (0x03/0x04) with quantity > 100 sustained over a 30-s window	zeek.modbus.function, zeek.modbus.quantity, source.ip	SCADA historian collection windows by source IP
6	Write to register addresses tagged as critical (setpoint, alarm thresholds) outside operator-approved write windows	zeek.modbus.function, zeek.modbus.start_address, @timestamp	Scheduled commissioning windows
7	Modbus request rate from one source IP exceeding 50 Hz over a 5-s window	source.ip, zeek.modbus.function (count aggregation), @timestamp	Load-testing source IPs during planned tests
8	More than 5 distinct unit_id values from one source IP within a 60 s window	zeek.modbus.unit_id, source.ip, @timestamp	Gateway hosts that legitimately query multiple unit IDs
9	More than 8 distinct function codes from one source IP within a 60 s window, with Modbus exception responses observed	zeek.modbus.function, zeek.modbus.exception_code, source.ip	Vendor diagnostic suites with cataloged source IPs
10	Any Modbus write function (0x05, 0x06, 0x0F, 0x10) with source IP outside the SCADA-poller allow-list	zeek.modbus.function, source.ip, destination.ip	SCADA poller source IP scoped to its authorised PLC set; misrouted writes still alert
11	Modbus read function from a source IP outside the read allow-list	zeek.modbus.function, source.ip	Engineering workstations during commissioning; read-only maintenance hosts
12	Same source IP issuing a read of register *R* followed by a write to *R* within a 30-s window	zeek.modbus.function, zeek.modbus.start_address, source.ip, @timestamp	SCADA poller’s known read–modify–write cycles
13	Sequential or strided reads spanning > 100 distinct register addresses from one source IP	zeek.modbus.start_address, source.ip	SCADA poller’s known register window
14	Failed logon events from one source against more than 5 distinct accounts within a short window	winlog.event_id (4625), user.name, source.ip	Service-account password-rotation windows
15	PowerShell child process matching known UAC-bypass module signatures (e.g., EnvBypassUAC)	process.parent.name, process.command_line	Known admin-tooling source hosts
16	gpupdate.exe spawned by cmd.exe or powershell.exe outside scheduled GPO refresh windows	process.name, process.parent.name, @timestamp	Scheduled GPO refresh windows
17	net.exe view or PowerShell Get-SmbShare from one host more than 3 times within a 5 min window	process.command_line, host.name	Backup hosts; known administrator workstations
18	New-ScheduledTask or schtasks /create via PowerShell from a non-administrator user	process.command_line, user.name	Admin user allow-list; software deployment service accounts
19	Rapid sequence of directory-listing commands (dir, ls, Get-ChildItem) from a non-interactive shell	process.command_line, process.parent.name	Indexing and search services
20	Read access to credential stores (e.g., %APPDATA%\Microsoft\Credentials, .ssh/id_rsa) by a non-system process	file.path, process.name	Backup agents; endpoint-security tooling
21	Truncation or deletion of ConsoleHost_history.txt or .bash_history	file.path, event.action	Scheduled cleanup tasks
22	Bursts of arp -a, net.exe view, or nbtstat -A from one host	process.command_line, process.name	Network-management workstations
23	reg.exe save HKLM\SAM or HKLM\SECURITY, or process patterns consistent with secretsdump.py	process.command_line, file.path	Known backup or imaging tooling
24	Read of HKLM\SOFTWARE\Microsoft\Windows NT\CurrentVersion\Winlogon AutoAdminLogon by a non-system process	registry.path, process.name	Domain-join provisioning tools
25	Bursts of net.exe user /domain, Get-ADUser, or dsquery user from one host	process.command_line	Ad hoc administrator queries from known workstations
26	New service creation via remote SMB (PsExec-style patterns) or admin-share usage from a non-administrator source	winlog.event_id (7045), source.ip, file.path (\\target\ADMIN$)	Known software-deployment and remote-management tools
27	More than 5 distinct destination IPs on destination.port = 502 from one source within a 60 s window	source.ip, destination.ip, destination.port	OT discovery tooling during commissioning windows

## References

[1] Bhamare D., Zolanvari M., Erbad A., Jain R., Khan K., Meskin N. (2020). Cybersecurity for industrial control systems: A survey. Comput. Secur..

[2] Formby D., Durbha S., Beyah R. Out of control: Ransomware for industrial control systems. Proceedings of the RSA Conference.

[3] Antonakakis M., April T., Bailey M., Bernhard M., Bursztein E., Cochran J., Durumeric Z., Halderman J.A., Invernizzi L., Kallitsis M. Understanding the Mirai botnet. Proceedings of the 26th USENIX Security Symposium (USENIX Security 17).

[4] Ullah I., Mahmoud Q.H. (2020). A Scheme for Generating a Dataset for Anomalous Activity Detection in IoT Networks. Proceedings of the Advances in Artificial Intelligence (Canadian AI 2020); Lecture Notes in Computer Science.

[5] Neto E.C.P., Dadkhah S., Ferreira R., Zohourian A., Lu R., Ghorbani A.A. (2023). CICIoT2023: A real-time dataset and benchmark for large-scale attacks in IoT environment. Sensors.

[6] Moustafa N. (2021). A new distributed architecture for evaluating AI-based security systems at the edge: Network TON_IoT datasets. Sustain. Cities Soc..

[7] Ghiasvand E., Ray S., Iqbal S., Dadkhah S., Ghorbani A.A. (2024). CICAPT-IIOT: A provenance-based APT attack dataset for IIoT environment. arXiv.

[8] Mirsky Y., Doitshman T., Elovici Y., Shabtai A. Kitsune: An Ensemble of Autoencoders for Online Network Intrusion Detection. Proceedings of the Network and Distributed System Security Symposium (NDSS).

[9] Al-Hawawreh M., Sitnikova E., Aboutorab N. (2022). X-IIoTID: A connectivity-agnostic and device-agnostic intrusion data set for industrial Internet of Things. IEEE Internet Things J..

[10] Ferrag M.A., Friha O., Hamouda D., Maglaras L., Janicke H. (2022). Edge-IIoTset: A New Comprehensive Realistic Cyber Security Dataset of IoT and IIoT Applications for Centralized and Federated Learning. IEEE Access.

[11] Sheeraz M., Durad M.H., Paracha M.A., Mohsin S.M., Kazmi S.N., Maple C. (2024). Revolutionizing SIEM Security: An Innovative Correlation Engine Design for Multi-Layered Attack Detection. Sensors.

[12] Alabdulatif A. (2025). A Novel Ensemble of Deep Learning Approach for Cybersecurity Intrusion Detection with Explainable Artificial Intelligence. Appl. Sci..

[13] Reis M.J.C.S. (2025). Edge-FLGuard: A Federated Learning Framework for Real-Time Anomaly Detection in 5G-Enabled IoT Ecosystems. Appl. Sci..

[14] Wazuh, Inc. (2026). Wazuh: The Open Source Security Platform.

[15] Elastic NV (2026). Elastic Security and the Kibana Query Language (KQL).

[16] Splunk Inc. (2026). Splunk Enterprise Security.

[17] Exabeam, Inc. (2026). Exabeam Fusion: SIEM with Behavioural Analytics.

[18] Alotaibi B. (2023). A Survey on Industrial Internet of Things Security: Requirements, Attacks, AI-Based Solutions, and Edge Computing Opportunities. Sensors.

[19] Yu Y.C., Ouyang Y.C., Lin C.A. (2025). PGTAD: Real-Time and Lightweight Multivariate Time-Series Anomaly Detection for IoT Using Patch Gate GRU Autoencoder. IEEE Access.

[20] Rahmani J., Detken K.O., Sikora A. (2026). An Integrated Testbed for MITRE-Mapped Attack Emulation in Industrial Control Networks. Sensors.

[21] Rahmani J., Sisinni E., Ferrari P., Detken K.O., Sikora A. (2026). Characterizing Domain Shift in PROFINET Intrusion Detection: A Multi-Site Ground-Truth Evaluation. Proceedings of the 22nd IEEE International Conference on Factory Communication Systems (WFCS).

[22] MITRE Corporation (2023). MITRE ATT&CK Caldera Framework.

[23] Cimino G., Deufemia V. (2025). SIGFRID: Unsupervised, Platform-Agnostic Interference Detection in IoT Automation Rules. ACM Trans. Internet Things.

